# Treatment Refractory Internalizing Behaviour Across Disorders: An Aetiological Model for Severe Emotion Dysregulation in Adolescence

**DOI:** 10.1007/s10578-020-01036-y

**Published:** 2020-08-03

**Authors:** Pierre C. M. Herpers, Josephine E. C. Neumann, Wouter G. Staal

**Affiliations:** 1 Karakter Child and Adolescent Psychiatry, University Centre, Reinier Postlaan 12, Nijmegen, 6525 GC The Netherlands; 2 Department of Psychiatry, Donders Institute for Brain, Cognition and Behaviour, Radboud University Medical Centre, Kapittelweg 29, Nijmegen, 6525 EN The Netherlands; 3 Department of Psychiatry, Radboud University Medical Centre, Geert Grooteplein 10, Nijmegen, 6525 GA The Netherlands; 4 Faculty of Social Sciences, Leiden Institute for Brain and Cognition, Postzone C2-5, P.O. Box 9600, Leiden, 2300 RC The Netherlands

**Keywords:** Emotion regulation, Attachment, Social anxiety disorder, HiTOP, *p* factor

## Abstract

Auto-aggressive behaviour, especially treatment refractory suicidality in adolescents with psychiatric disorders, may be challenging to clinicians. In search of therapeutic possibilities, we have integrated current opinions regarding causality and interdependency of suicidality and auto-aggressive behaviour across disorders within the HiTOP framework. We propose a developmental model regarding these unsettling behaviours in youths that may help to guide future directions for research and interventions. We argue that the interdependent development of biologic factors, attachment, moral reasoning and emotion regulation in an overprotective environment may lead to social anxiety and later during development to emotion dysregulation and severe internalizing behaviour disorders. To optimize treatment efficacy for both internalizing and externalizing behaviour, we emphasize the importance transdiagnostic interventions, such as addressing non-compliance, restoration of trust between parents and their child, and limitation of avoidance behaviour. These may be seen as higher order interventions within the HiTOP framework.

## Introduction

Mechanisms of persistent self-destructive behaviour in patients with internalizing behaviour problems are still poorly understood. Although a vast body of research exists with results at a variable level, research regarding pathway approaches seems to be relatively sparse, yet important to understand individual development of psychopathology [1]. This type of research may be hampered by current diagnostic systems, such as DSM-5 [2] or ICD-10 [3] that aim to classify taxonic conditions and aim to be a-theoretical regarding origins. However, many psychiatric disorders do not show to be clearly demarcated taxonic entities [4, 5], and may be criticized because of it’s a-theoretical nature [6]. Hence, different systems are being developed to diagnose and investigate dimensional entities. One of these is the Hierarchical Taxonomy of Psychopathology (HiTOP; [7]). HiTOP aims to combine individual psychopathological phenomena into homogeneous domains, and grouping them into psychopathology spectra (e.g., internalizing, externalizing, and thought disorder). As such, HiTOP has a strong focus on quantitative nosology, clinical phenomena and a hierarchical structure. It supports a transdiagnostic dimensional approach [8] and aims to facilitate reconceptualization of psychopathology by accentuating their interdependence and relevance for one another [9].

HiTOP is highly driven by grouping clinical psychopathological phenomena into a meaningful hierarchical structure. The HiTOP spectra correlate with the overall liability of a person to develop psychopathology, the *p* factor [10]. This liability may lead to psychopathology in one of the spectra. The *p* factor theory underscores the importance of genetic underpinnings for the general liability to develop psychopathology, although transdiagnostic mechanisms may also be found at different domains, such as poor emotion control, or different levels of thought disorder [11]. A psychopathologic phenomenon in which poor emotion regulation and disturbed thinking may be explicitly present is suicidality. Moreover, it has been related to a range of psychiatric disorders as a transdiagnostic factor based on a general psychopathology liability [12]. However, the *p* factor is a *general* construct which may be difficult to translate to the individual patient, because variance at a group level needs to be critically studied at an individual level for better understanding of diversity in process and outcome [1]. As such, HiTOP does not yet incorporate aetiology [13], neither on a group level nor on an individual level.

Aetiology is an important topic in understanding disorders and, as such, diagnosing psychopathology. As HiTOP seems to lack a clear statement on aetiology, the question remains on how to understand aetiology of apparently distinct disorders yet showing overlap in their presentation. Suicidal behaviour, including suicide ideation as well as parasuicidal acts, has been associated with complex developmental pathways [14], and covers a broad spectrum of psychiatric conditions [15–17]. In adolescence it is often associated with depressive disorder [18] and with borderline personality disorder (BPD), and may be challenging to treat [19].

Aiming to address aetiology as well as emotion dysregulation in these disorders, several psychotherapeutic interventions have been developed. Two interventions that seem to be the most important of these are Mentalisation Based Therapy (MBT; [20]) and Dialectical Behaviour Therapy (DBT; [21]). MBT is a psychoanalytically oriented intervention that makes mentalizing a core focus of therapy [22]. Mentalizing is the cognitive process by which we make sense of each other and ourselves, in terms of intentional states [23, 24]. Traditional MBT provides individual and group sessions, crisis planning and integrated psychiatric care. Individual sessions focus on developing a therapeutic alliance through a close attachment relationship and on maintaining an optimal level of arousal during interactions with others by addressing the details of the mentalizing process. Group sessions focus on mentalizing in a more complex interactional process [23]. The importance of addressing attachment, and epistemic trust has been stressed by MBT [25]. In contrast, DBT has emerged from standard behaviour therapy and developed to a transdiagnostic therapy for clients with complex, high-risk disorders [26]. It is a modular and hierarchical treatment consisting of a combination of individual psychotherapy, group skills, training, telephone coaching, and a therapist consultation team. Although DBT has a strong focus on acceptance, it provides a hierarchy of what to treat and when to treat it for a particular patient. DBT consists of four stages: (a) decrease dysfunctional behaviour, (b) experience emotion, (c) reduce ordinary problems in life, and (d) increase a sense of completeness. Treatment is split into two domains: (a) skills training and (b) problem solving and motivation [26].

For adolescents, the evidence of efficacy for both DBT and MBT is promising, although somewhat limited [27–29]. Other psychotherapeutic interventions, such as emotion regulation therapy, cognitive analytic therapy, and cognitive behaviour therapy show at best modest improvement [27, 28]. Furthermore, improvement during psychotherapeutic interventions seem to decrease at follow-up [30]. As such, a substantial group of these adolescents show to be treatment refractory [30, 31], and may even show to be determined not to respond to treatment [32].

As non-response to psychotherapeutic interventions may be due to neurobiological factors, pharmacotherapy may be described to patients with emotion dysregulation. However, pharmacological interventions in adults appear at best adjunctive to psychotherapeutic interventions [33], and the evidence regarding the efficacy of pharmacotherapy is sparse. Therefore, it has been recommended to avoid pharmacotherapy in BPD. Whereas many scholars focus on improving efficacy of current treatment modalities, information regarding the underpinnings of treatment refractoriness remains scarce [34].

Another reason for non-response may be that the underlying condition of treatment refractory suicidal behaviour can be difficult to diagnose, and one may diagnose a disorder that is not actually present [32]. From a perspective of emotion dysregulation (e.g., borderline personality disorder) it may be difficult to diagnose because of transient changes in symptomatology [35]. From a perspective of thought disorder (e.g., autism), it may be difficult to diagnose because of difficulties in identifying behaviour problems correctly [36]. This may further be complicated because of the presence of symptomatology that, at a behavioural level, seems to correspond with different kinds of psychopathology, such as internalizing and externalizing behaviour problems (e.g., [37, 38]), as well as well as to disorders in the thought disorder spectrum (e.g., [39]). Difficulty to diagnose specific disorders may also be due to the cross-disorder presence of different types of internalizing behaviour, such as anxiety, depressive symptoms, somatization, eating disorder, and emotion regulation disorders [40].

From a DSM perspective, treatment refractory suicidal behaviour may present itself in a range of disorders with or without comorbidity. HiTOP aims to take a different perspective. The HiTOP framework describes developmental factors that moderate development of psychopathology, and aims to gather data to inform regarding natural course and treatment efficacy. It does not describe specifically how these factors interact in order to develop a specific disorder or comorbidity. Furthermore, it does not seem to address a possible time line in the development of disorders that may precede or follow each other. HiTOP implies that comorbidity may possibly reflect higher order defect, implying genetic differences more prominently than environmental differences.

Treatment refractory suicidal behaviour can be found in a range of disorders in which suicidal behaviour predominantly has been labelled as internalizing behaviour problem. Therefore, we broaden our focus from treatment refractory suicidal behaviour to *treatment refractory internalizing behaviour* (TRIB), which we define as internalizing behaviour based on emotional developmental pathology, having shown treatment refractoriness to guidelines informed treatment in previous mental health services. The reasons for this treatment refractoriness seem to be poorly understood. The current paper will discuss clinical difficulties in TRIB, with respect to diagnostic considerations and classification, and with respect to therapeutic issues. We performed literature searches in the PubMed database regarding the specific subject in this essay. In our literature searches we aimed to find meta-analyses, structured reviews, papers written by leading scholars to found our theory. As such, we based our theory on best available evidence.

We argue that a focus on ‘diagnosis by classification’ may distract attention of the underlying cause of the core problem, and consequently lead to inadequate choices for interventions. We will introduce a theoretical framework on TRIB. In this model, we reconsider social anxiety as a broader concept and state that the development of early developmental social anxiety may be seen as a central pathway to consecutive internalizing behaviour disorders in adolescence. In line with MBT and DBT, we address issues as trust and motivation for treatment that may be explicitly lacking. However, where MBT and DBT predominantly seem to focus on the individual, we stress the importance of intensively involving parents for improvement of emotion regulation and further development. The TRIB model implies similarities between juvenile emotional disorders and juvenile disruptive behaviour disorders (DBDs). With this model, we aim to provide a hypothetical explanation for TRIB, thus providing a guiding framework for interventions and future research.

## Diagnostic and Therapeutic Issues Regarding TRIB

Having discussed TRIB in relation to diagnoses from a HiTOP perspective and in relation to important psychotherapeutic interventions, it remains difficult how to translate a model like HiTOP into a system that is useful for mental health workers [9]. Therefore, it seems important to discuss issues that relate to clinical aspects of TRIB.

### Thought Disorder Versus Emotion Dysregulation

In clinical practice, the key question is not typically “to treat or not to treat?”, but rather, “what level of intervention best suits this level of need?” [41]. As such, disorders that currently may be seen as distinct, may be better conceptualized as specific manifestations of the same condition [42]. To illustrate this perspective, we will focus on two disorders which are characterized by either (a) predominantly internalizing and externalizing behaviour (i.e., BPD), or (b) predominantly thought disorder (i.e., autism spectrum disorder; ASD [2, 43]). Although both disorders apparently represent different entities, both may show severe emotion dysregulation with self-destructive behaviour, and may show overlapping signs and symptoms, such as psychotic disorders [43], and aggressive and suicidal behaviour [44], eating disorders [45] and anxiety disorders [46].

Both disorders show social communicative inflexibility [2, 47]. This may be due to neurobiological impairments in information processing [48] as well as to emotion dysregulation [49]. This may imply that all behavioural problems in youth with ASD should be viewed as the result of the neurobiological impairments that underlie ASD, leading to the tendency not to diagnose any comorbidity in case of ASD [50]. Nevertheless, behavioural problems in youth with ASD may stem from emotion regulation disorders as well. Therefore, we argue that a subgroup of patients with ASD, ASD should be seen as a complicating factor in emotion regulation.

Emotion regulation is a capacity that every individual needs to master in order to function successful in society, disregarding any disability, being it physical, intellectual or mental. As such, the tendency to treat a classification may deflect from diagnosing phenomena in a way that might me more helpful. Such a deflection implies delay of adequate diagnosis of TRIB resulting in decreased effectiveness of potentially adequate therapies. Moreover, the severe presentations of internalizing disorders may be the result of delay in adequate action/treatment, or even inadequate action/treatment. This perspective may, at least in part, explain findings that show the (cumulative) role of life events prior to the occurrence of depressive disorders in adults [51].

### Internalizing Versus Externalizing Behaviour

A specific diagnostic aspect that puzzles clinicians is the overlap between internalizing and externalizing behaviour disorders. Correlations between internalizing and externalizing symptoms have been found to be high and comorbidity seems, in line with HiTOP, to be the result of shared, overarching psychopathological processes [42, 52]. These overarching processes may not only be the result of biologic processes (see [42]), but also of psychological processes. As those adolescents that have poor problem-solving skills are more likely to experience suicide ideation under stressful conditions than other adolescents [53], suicidal behaviour and deliberate self-harm in adolescents could be explicitly related to poor problem-solving skills. Thus, suicidal behaviour appears to present itself in relation to negative emotions. As such, suicidal –and related– behaviours seem to be the result of poor emotion regulation skills.

Poor emotion regulation skills are also seen in DBDs and there is discussion going on to which extent auto-aggression is similar to hetero-aggression. Importantly, DBDs (externalizing behaviour problems) and BPD (internalizing behaviour problems) are thought to have shared aetiology, yet leading to a gender related outcome. Circumstances in early life have been found to be similar, that is, high-impulsivity and high-risk environments, but then girls may develop in a more internalizing direction in the form of BPD and boys in a more externalizing direction in the form of antisocial personality disorder (ASPD) [54]. Nevertheless, in BPD not only internalizing behaviour can be found, but externalizing behaviour as well [55]. In addition, recent research suggests the major difference between BPD and ASPD does not seem to be aggressive response generation, but prosocial and avoidant response generation. BPD has been found to be more related to increased avoidant or prosocial responses, whereas in ASPD the contrary has been found [56]. Furthermore, inadequate emotion regulation may be reinforced by non-effective parenting styles [21]. These findings raise questions whether both disorders are different from each other in their underlying origins or represent a different expression of the same underlying condition. Moreover, these findings imply that psychopathology such as suicidality, deliberate self-harm and social anxiety should be seen less as internalizing, and more as externalizing behaviour than they are considered to be up till now.

### Lack of Motivation for Therapy

Treating youths with auto-aggressive behaviour remains challenging. To reduce self-harm, it seems important to develop interventions that aim to improve self-regulation of emotion and decision making and thus on reducing rash reactivity to emotions [57]. As mentioned already, there are several, well documented cognitive behavioural treatment modalities available (e.g., DBT, MBT) that show promising results. However, not all patients show (full) recovery. Some even deteriorate, which may result in either voluntary or involuntary admissions in somatic or psychiatric hospitals. During hospital admission, treatment modalities regarding TRIB usually focus on relieving negative emotions such as anxiety and depression, based on principles of providing medical care. However, further deterioration instead of improvement often occurs [58].

Motivation is an important non-specific factor in therapy [59], which seems to be accountable for non-effective therapeutic interventions, and low treatment fidelity [60]. This lack of motivation seems to be driven by frustration, that is, a mix of anger, anxiety, depressed mood, and feelings of helplessness and hurt feelings, eventually leading to dichotomized thinking expressed as suicidality (“If I can’t have things perfect in my life, then I don’t want to live at all!”). As lack of motivation seems to be a central topic, and essential for the extent of improvement one can reach, it is important to discuss whether this should be seen as (a) an involuntary result of disease, and thus treated like this, or (b) the result of a voluntary decision, even though it may not seem to be wisely taken.

Low intrinsic motivation for treatment may be associated with decreased feelings of competence, autonomy and relatedness [61]. To increase motivation for interventions, therapists need to be communicate in an empathic and supportive manner [62]. These attempts include interventions, such as providing confidence, reassurance, comfort, that often have been applied by parents as well. Nevertheless, in youth with treatment refractory auto-aggression, these types of interventions may increase feelings of demoralization and helplessness in both patient and parents. Moreover, demoralization and helplessness may be the result of the interventions (admission and concomitant care) itself. The result is that not only the patient, but also parents and hospital staff feel helpless, leading to increased demoralization the longer a patient stays in the hospital. In such cases, offering medical interventions with a focus on providing care only may not only be redundant, but may lead to iatrogenic damage, as providing care may lead to reinforcement of adverse behaviour.

### Limited Efficacy of Biological Interventions

Treatment refractoriness of these patients refers not only to psychotherapeutic, but also biological interventions. The efficacy of antidepressants in adult depression has been established [63]. In contrast, the efficacy of medication in adolescents is less clear [64]. Findings regarding the efficacy in relation to severity of depression are inconsistent [65–67]. At best, antidepressants may speed up improvement in the early phases of treatment, and may prevent relapse [68]. Partly, this could be explained because in adolescents, antidepressants were found to be differentially effective for different disorders (i.e., strongest for non-OCD anxiety disorders, intermediate for OCD, and more modest in major depressive disorder (MDD) [69]. The efficacy of antidepressants in social anxiety disorder (SAD) is not related to initial severity of symptoms [70]. For both depressive and anxiety disorders, combination therapy of an antidepressant with CBT may be statistically more effective [71].

In BPD, the efficacy is limited as well. Drugs for BPD may take the edge off symptoms but do not lead to remission of the disorder. Moreover, as all agents seem to have similar effects, there is little logic in prescribing polypharmacy regimes [72]. The fact that aggression is seen as the consequence of a specific underlying psychiatric disorder [73], and not as expression of emotional dysfunctional development may explain limited effectiveness of medication. Furthermore, medication for oppositional or aggressive behaviour is not the first-choice treatment, whereas caution is needed for the use of antipsychotics because of potentially serious side effects and suspected long-term developmental risks [74]. Hence, though medication may show significant positive effects, there remains a substantial proportion of patients with residual or full-blown emotion dysregulation, requiring subsequent intervention steps (cf. [75]).

As non-response comprises an important issue, there is a search for other biological therapeutic interventions. Electroconvulsive therapy (ECT) may be seen as a safe and effective modality for treatment-refractory psychiatric disorders in adolescents as in adults [76]. Possible effectiveness has been shown for depression in youth [77], and non-suicidal self-injury in female adolescents [78]. However, there are no published randomized controlled trials of ECT yet [76, 78]. A review regarding repetitive transcranial magnetic stimulation (rTMS) for adolescents with treatment-resistant depression reports that rTMS might have some benefit for these youths. However, all reviewed studies were still either case-reports or open label [79]. Evidence for effectivity of rTMS in anxiety disorders adults is inconclusive, at best weak [80]. To the best of our knowledge, evidence on efficacy of rTMS in anxiety disorders in youths is lacking. In adults, the possible therapeutic effects of ketamine [81] and psychedelics [82] have been investigated increasingly. However, for adolescents we could find only one case report on ketamine [83].

Efficacy of medication may also be limited because of a *nocebo* effect [84]. Generally, the term nocebo effect is used when an inert substance causes perceived harm [85]. We would like to broaden this definition with the possibility to perceived neutralization of an active substance. Importantly, antidepressants are generally associated with high nocebo effects, which may be moderated by higher education, hypersensitivity to medications, less experienced physicians, female gender (for a review, see [86]), whereas it still is unknown to which extent a lack of motivation for (pharmaco)therapy may neutralize beneficial effects of medication. Furthermore, recent research shows that the effect of antidepressants may be dependent of the level of environmental stress [87]. Because treatment refractory youths with BPD experience severe stress to any social responsibility, and severe avoidance behaviour, this may be an important reason as well why antidepressants only show limited effectiveness.

## A Preliminary Aetiological Model for TRIB

Research findings imply that emotional behaviour disorders, such as anxiety, depressive symptoms, somatization, eating disorder, and emotion regulation disorders may be seen as ineffective coping [88], that is, strategies that aim to cope with difficulties in life, but have a detrimental effect. As such, there may be similarities with auto-aggression. Therefore, we described suicidal behaviour as pars pro toto example to illustrate that TRIB, in which suicidal behaviour as well as other pathological internalizing behaviour, may be seen as the result of inadequate coping in emotion regulation. The efficacy of both psychotherapy and psychopharmacology in the treatment of internalizing behaviour problems has its limitations. Thus, there remains a substantial proportion of patients with residual or full-blown symptomatology, requiring subsequent intervention steps.

The HiTOP framework may be helpful in findings ways to increase therapeutic efficacy by reconceptualizing the psychopathologic phenomena. If comorbidity is due to higher order mechanisms, then we have to search for higher order answers regarding origins and therapeutic interventions. This implies a search for factors that moderate the *p* factor. Although the *p* factor has been primarily related to genetic origins of psychopathology, it does not rule out developmental influences that may lead to psychopathology. And if the *p* factor indeed is related to a genetic liability to develop thought disorder [11], how may environmental factors, such as parenting affect developmental deviations?

### Interaction Between Genes and Environment Regarding Attachment, Moral Development and Emotion Regulation

HiTOP states that genetic factors play an important role in neurobiological and neuropsychological development, which may lead to entities that are believed to have strong biologic underpinnings, such as, for example, intelligence [89], or ASD [90]. Nevertheless, environmental factors are important as well. Especially TRIB disorders have been related to the interplay of attachment, moral development and emotion regulation. Attachment processes have been closely linked to moral development [91] and emotion regulation [92]. Biological underpinnings have been described for the development of attachment [93], moral reasoning [94] and emotion regulation [95], and starting in early infancy [96, 97]. Research suggests important links between early caregiver–infant attachment and health related physiological processes (e.g., stress) and resilience (e.g., the capacity for managing stress-related metabolic demands) [98]. Epigenetic effects have been reported as well [99]. As such, environmental factors, such as parenting and social interaction with others may moderate the process of biological anchoring.

Biological anchoring in the brain is a process that takes place when specific behaviour is activated by repetition. Biological changes take place through which external stimuli lead automatically to predetermined reactions. These mechanisms have been described for behaviour problems [100] and for non-suicidal self-injury [101]. Furthermore, quality of parenting and stress moderate hormone systems which in turn moderate secure attachment [102], while habituation reduces the effort required for future engagement in emotional reactions and behaviour, and thus to change ones behaviour (cf. [101]). Hence, moral development, emotion regulation and attachment are moderated not only by biological factors [95], but by environmental factors—that may lead to biological changes—as well. Attachment, moral reasoning and emotion regulation do not develop isolated, but intertwined [103]. Moreover, these processes are moderated by behavioural principles as formulated by learning theory, that is, classic and operant conditioning [104].

### Attachment, Moral Reasoning and Emotion Regulation

Parenting style, and the resulting interaction starts in early life and is stored as a conceptualization of what to expect in relations, that is, attachment [105]. Attachment processes have been proven to play an important role in child development [106], and may best be described as script-like representations of secure base experience mirroring the extent to which children feel supported by their parents, especially in times of stress [107]. Attachment security has been related to parental reflective functioning [108]; attachment scripts have been found to be transferred intergenerationally [109] and show longitudinal stability [110]. A secure base script may develop when consistent and coherent support has been provided in early childhood [107]. If the establishment of a secure base fails, effects may be long lasting, leading to increased vulnerability to feel disappointed and rejected. This may lead to insecure attachment styles [111, 112]. As such, insecure attachment has been thought to be an important transdiagnostic factor in the development of psychopathology [113].

Other important transdiagnostic factors may be found in moral reasoning and emotion regulation. As mentioned before, attachment processes are closely related to moral reasoning and emotion regulation. Attachment develops through learning processes, resulting in internal working models, and thus in either secure or insecure base scripts [114]. The development of moral reasoning is a process starting in early life [96], continuing into adulthood [115]. Predominantly based on the seminal work of Kohlberg [116], moral development has been conceptualized predominantly related to the extent in which children and youth internalize and comply with adult and societal rules and requests [96]. As such, principles from learning theory already play an important role through which moral development can be seen as the internalizing of parental moral reasoning into internal working models. Emotion regulation during development is strongly linked with neuropsychological functioning (e.g., [117]), as well as environmental factors, especially transference of emotion regulation by parents [118]. Through their parents, children learn to mentalize their emotions and thus, to handle them. [91].

**Fig. 1:**
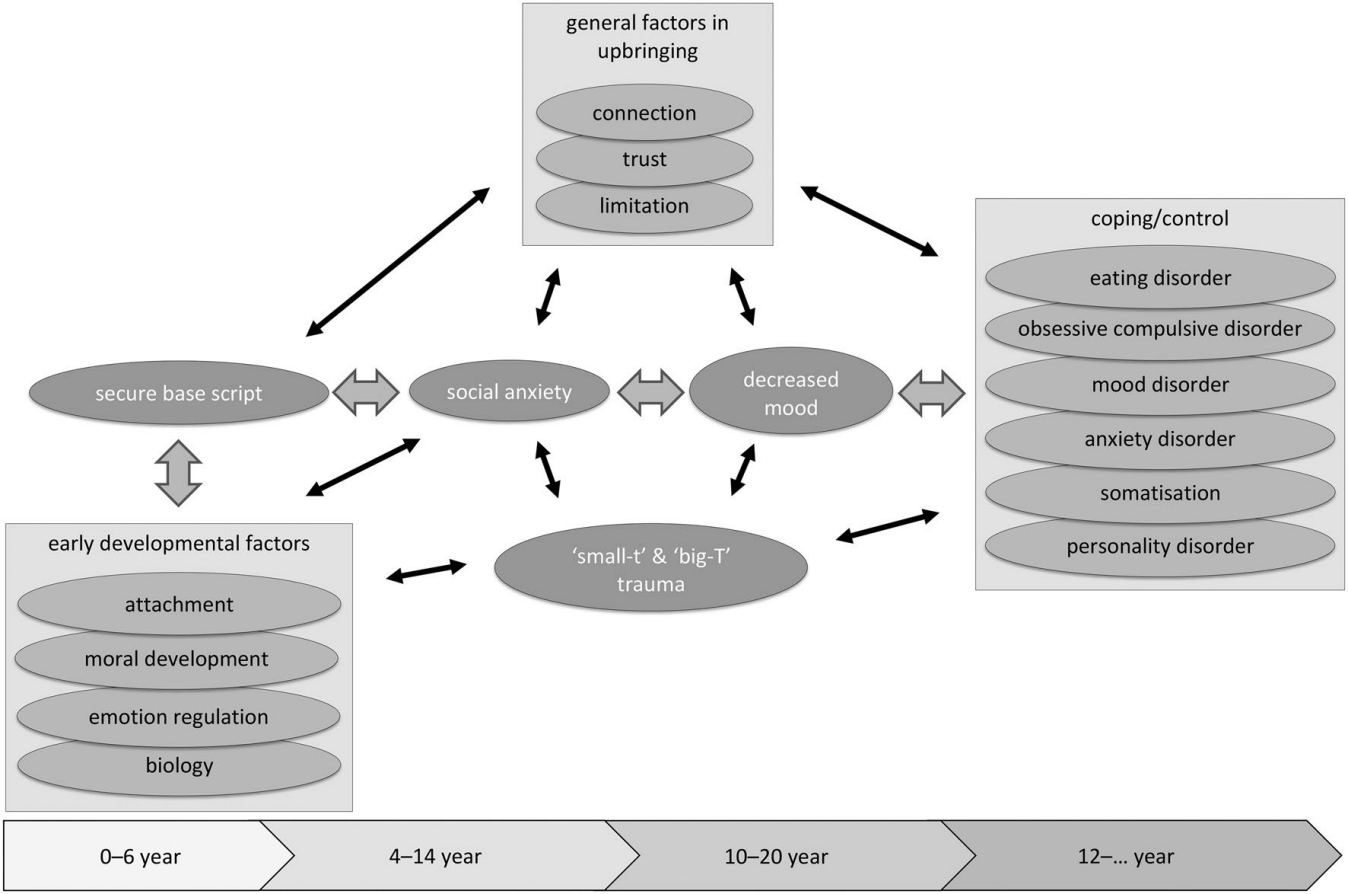
Aetiological model for therapy refractory internalizing behaviour

In sum, attachment, moral reasoning and emotion regulation show interactive moderating roles and are internalized through learning processes into internal working models. The level of moral development may have an impact on how emotional disturbances are mastered. The neurobiological make-up of a child and the interaction with (i.e., reaction of) parents may moderate which of the basic emotions a child will be expressed and reinforced. Especially when specific behaviour is intermittently reinforced in early childhood, it may become highly resilient to extinction [114]. The result will be that it is difficult to find new strategies in later phases of life. Through these interdependent processes, specific patterns of attachment, moral reasoning and emotion regulation may become dominant in a way that may lead to psychopathology (see also Fig. 1).

### Parenting

Parenting is an important environmental factor that moderates emotional information processing [108]. It is a complex activity because it requires to flexibly deploy different practices or strategies in various situations in a way that similar practices may have different meanings depending on children’s developmental status and cultural context [119]. Adequate parenting plays an important role in mentalizing (i.e., providing informative and helpful meaning to emotions), thus helping the child to contain its emotions. As such, autonomy-supportive parenting plays an important role in fostering adequate behaviour regulation [120]. Yet, at many parent and child levels things can go wrong in the intergenerational transfer of emotion regulation and prosocial behaviour [25]. Parental emotional distress may reinforce internalizing behaviour problems in their child (cf. [121]), because they (unintendedly) support their child’s avoidance behaviour. As such, TRIB may be the result of unsuccessful limit setting behaviour by parents in early childhood. In combination with pleasing behaviour of the child because of insecure attachment, this may lead to emotion dysregulation [122], and thus to social anxiety (cf. [123]). In line with these findings, parent-dependent chronic adversities have been found to be significantly related to more severe child anxiety symptoms [124]. Also, internalizing behaviour in children has been found to be related to psychological control by their parents [120], whereas social anxiety has been found to be related to overprotective parenting [125].

### Social Anxiety Disorder

Social anxiety, often described as shyness [126], or perfectionism [127] without explicit impairments often precedes social anxiety disorder (SAD). In general, the core symptom of SAD is believed to be a “marked fear or anxiety about one or more social situations in which the individual is exposed to possible scrutiny by others” [2]. Youth high on social anxiety often show avoidance behaviour, and biased attention and interpretation of social information [128]. SAD is one of the most prevalent mental disorders [129] and most often presents in childhood or early adolescence [126]. When SAD is present with comorbidity, prognosis worsens [130]. As such, SAD may have great impact on personal and social functioning [131].

Although SAD may have great impact on daily life, it is often underrecognized, whether with or without comorbidity [131]. An important reason for this may be that patients avoid talking about their social anxiety [129]. Another reason may be that DSM criteria focus on criticism of others, whereas SAD may comprise self-criticism as well [132]. Therefore, it may be more helpful to define SAD as a “persistent fear of or anxiety about one or more social or performance situations that is out of proportion to the actual threat posed by the situation” [131]. This definition provides room for the notion that a patient with SAD may not only be afraid of criticism of others, but of himself as well. Yet, self-criticism may also be defined as perfectionism [127]. As such, the overlap between the concepts of SAD and –maladaptive– perfectionism is large. Therefore, we believe that broadening the definition of SAD may increase recognition.

Increasing recognition is important because of overlapping symptomatology with separation anxiety disorder (separation AD) and generalized anxiety disorder (GAD). Although these anxiety disorders have been associated with insecure attachment [133, 134], less is clear about how disturbed attachment may lead to different types of anxiety disorders. Separation AD may be seen as a disorder in which attachment developed more problematic than in SAD, and thus addresses more basic fears (of ‘being left alone and unable to survive’). On the other hand, it may be seen as a consequence of more severe SAD, that is, a hyper-focus on a specific aspect of social anxiety (‘being left alone because no-one likes me’). In a similar vein, we see GAD as a further broadening of social anxious worrying to a general pattern of worrying about ‘everything’ one doesn’t control. However, this hypothesis needs further testing. Nevertheless, we argue that in at least a part of adolescents with TRIB, unrecognized SAD may play an important moderating role in the development of more severe psychopathology.

This statement finds it support in studies showing increased comorbidity between social anxiety and BPD [135], and that social anxiety plays a moderating role in adult depression [136]. Furthermore, maladaptive perfectionism in primary school children has been found to be related to increased levels of internalizing as well as externalizing behaviour [137], and precursors of SAD, such as self-criticism and avoidance behaviour may be reinforced by parenting [129]. Moreover, perfectionism has been found to moderate decreased feelings of belongingness and suicide ideation in adolescents [138]. Hence, we state that increased social anxiety may lead to perfectionism, and thus may moderate avoidance behaviour, moral reasoning and emotion regulation.

### Loss of Trust

This leads us to a next step in our theory: If both internalizing and externalizing behaviour problems can present simultaneously, should these be seen as different entities, or one entity with different expressions? We argue that both internalizing and externalizing behaviour problems may be seen as avoidance behaviour, through which a child aims to avoid/master distress. As such, both behaviours may be seen as expressions of emotion dysregulation in which the youngster has stopped seeking support with his parents in times of distress, because of previous interpersonal disappointments and loss of trust [92]. Hence, when regular coping mechanisms in SAD hamper, mood levels may decrease [139], leading to suicidal ideation and deliberate self-harm in an attempt to dampen emotions and relieve stress. As this kind of avoidance behaviour does not help, anger and hopelessness may increase even further. When this happens, suicidality is around the corner in a combination of internalizing and externalizing behaviour, and both loss of trust and meaning of life [140, 141].

### The Role of Trauma

Posttraumatic stress disorder, as defined in DSM-5, requires the exposure to actual or threatened death, serious injury, or sexual violence [2]. However, not only ‘*big-T trauma*’. such as natural disaster, life threatening accident, acts of war or violent personal assault may lead to posttraumatic stress. Adverse childhood experiences (ACEs), such as abuse, neglect, family instability, parental mental illness, parental substance abuse, parental incarceration, domestic violence, and neighbourhood violence may often be categorized as big-T trauma as well [142]. Exposure to ACEs has been found to be strongly associated with the development of externalizing and internalizing behaviours in—young—adolescence [143].

In contrast, *small-t trauma* refers to experiences that may seem negligible to an adult, but extremely important to a developing child [144], such as not feeling understood, heard or helped, or feeling teased at stressful moments. These moments may be labelled as stressful interpersonal experiences representing mildly unsupportive single learning events that negatively moderate caregiving experiences [114]. Stressful interpersonal experiences may lead to emotional dysregulation as well [145] and relational victimization has been found to be related to increased suicidality in adolescent females [146]. Traumatization in parents may lead to parenting limitations which can disrupt development of the child [147]. As such, emotion dysregulation may be the result of direct or indirect, and big-T or small-t trauma which can occur at any time in life and development, thus disturbing attachment, emotion regulation and moral reasoning.

## Implications for Therapy

In line with the HiTOP framework [8, 9], we outlined an aetiological model for TRIB from a transdiagnostic dimensional approach, addressing the interdependence of biologic and psychological developmental, and specific environmental aspects in relation to the development of psychopathology. As such, the TRIB model may closely resemble the underlying theories of MBT (with an emphasis on attachment) and DBT (with an emphasis on emotion regulation). However, both therapeutic interventions originally have been developed for adults, and focus strongly on individuals. Furthermore, even with pre-therapeutic motivation enhancing interventions, MBT and DBT may not always be feasible for youth with TRIB because of lack of motivation for—either individual or systemic—therapy. In these patients, it seems to be important to relocate attention towards three transdiagnostic principles, that we believe to be underrated, though most important in this specific group of patients. We link these principles to general principles in upbringing: (a) making connection, (b) giving trust, and (c) setting limits to behaviour. In a therapeutic context, these principles may be translated as transdiagnostic factors in therapy: (a) addressing non-compliance, (b) restoring trust, (c) addressing avoidance behaviour.

### Addressing Non-compliance

Non-compliance is an overall, cross-disorder problem in healthcare, leading to increased medical healthcare and related costs [148]. This also seems to apply for TRIB. Even though treatment interventions are increasingly effective, there remains a subgroup of patients with TRIB that shows demoralization and low motivation for treatment. Moreover, these youngsters may also show low or even absent compliance by not following the agreements they made in treatment, or actively hindering treatment. It is important to increase the recognition of the patient’s role in healthcare and why he/she does not adhere with treatment principles [148].

Non-adherence may be the presentation of avoidance behaviour, and thus may have an interactional meaning. If psychiatric symptoms are being used in interaction to pressure the environment to do things according the wish of the patient, there is a parallel with disruptive behaviour disorders, in which it is often very difficult for parents to limit their child as well. In these cases, following, for example, the NICE guidelines for DBDs [149], with a strong emphasis on evidence-based psychosocial treatments [150] may be better in place than guidelines for suicidal behaviour. More specifically, as parental cognitions are crucial to engagement [151], it is important to incorporate general approaches to improve families’ engagement in treatment, such as brief early treatment engagement discussions, family systems approaches, enhancing family support and coping, and motivational interviewing [152].

Moreover, parental engagement—that is, both mothers and fathers [153]—in treatment seems to be of utmost importance in all child and adolescent psychiatric disorders [150], whereas engagement seems to be one of the most critical factors to obtain positive treatment results in treatment of youths and adolescents [154], and parents [155]. In seemingly contrast, in meta-analytic studies relationships between general therapeutic relationship/alliance measures (to youth, parents, or family members) and treatment outcome appear to be small to moderate [154, 156]. This might be due to bias: if one is not motivated for treatment, one may be excluded at some point in the study. Nevertheless, the quality of the therapeutic alliance plays an important moderating role in treatment processes [157], and therefore needs to be addressed.

### Focus on Systemic Interventions to Restore Trust Between Patient and Parents

A transdiagnostic factor such as hopelessness about belongingness has been found to play an important role in suicidal desire [158], and treatment adherence has been found to be related to positive family attitude towards treatment [159]. This may, in part, clarify why the evidence for impact of psychotherapeutic interventions on self-harm seems to be small [160]. A trustful attitude may have a positive moderating effect on treatment efficacy. An increased focus on attachment processes might clarify the development and usefulness of transdiagnostic treatment interventions, especially when focusing on underlying forms of dysfunction that specific disorders bare in common [161], such as emotion dysregulation [162]. The effectiveness of such interventions has been shown in youth with depression, anxiety, autism and conduct problems [163–165]. This is in line with the contention that effectiveness of DBT in internalizing as well as externalizing disorders may be based on a common underlying dysfunction in emotion regulation [166]. thus providing ‘central’ therapeutic elements for several disorders, integrated into one modular protocol. Results for such therapeutic interventions are promising in the short term [167], as well as after 2-year follow-up [168]. However, if early developmental processes, such as attachment, emotion regulation and moral reasoning play an important role, then inter-generational transmission of these processes may play an important role as well. Therefore, it is important to focus on a systemic approach to TRIB. Attachment Based Family Therapy (ABFT) [113], and Multisystemic Therapy (MST) [169] are systemic intervention techniques that have been shown to be effective [170].

When applying a systemic approach, pathways to reach improvement may comprise: (a) restoration of communication and trust between adolescent and parents [113], (b) empowerment of parents and their child [171], and (c) learning to accept one’s own thoughts and feelings and being able to endure them [166, 172]. Several therapeutic interventions, such as ABFT, MST, DBT, MBT and empathic-emotion recognition training [173] now seem to be applied across disorders.

### Addressing Avoidance Behaviour

A central theme in treatment of TRIB is the management of avoidance behaviour. If TRIB should be seen as an attempt to avoid societal responsibilities in life, any therapy has to focus on decreasing avoidance behaviour. Operant conditioning, in which consequences of behaviour are highlighted and changed, seems to be preferable. Many of the therapeutic guidelines may show overlap, but some implications of these guidelines seem to be important for daily clinical practice. These implications concern the importance of no or only short-term hospital admission [174], mandatory rooming-in of parents, collaboration of every involved healthcare provider, and focus on behaviour that can be changed.

Positive effects of admission of youth with TRIB often last only for a few days, which seems to be in line with the finding that long stay hospital treatment for BPD has been shown to lead to deterioration [58]. Result of long stay hospital treatment may be the recurrence of crisis behaviour, deliberate self-harm and suicidal acts, which leads everybody to feel powerless. This may induce a wish for continuation of the admission. Yet, the importance of short-term hospital admission may lie in the fact that youth with TRIB often show avoidance behaviour regarding daily life societal responsibilities. In the short term, hospital admission leads to relief of feelings of stress, thus reinforcing avoidance behaviour. As such, feelings of powerlessness, hopelessness and avoidance behaviour may lead to a decrease of motivation for therapy.

Intrinsic motivation for therapy will increase only when a patient starts to feel the power that he/she will be able to manage his own feelings again. For this, patients, and their parents, need to be motivated to agree on the tasks they have to carry out in order to achieve treatment goals [175]. Hence, it is important to discuss whether patients feel confident they are able to change. As such, it is important to change the perspective of providing therapy: instead of searching for underlying causes of crises it is important to empower patients in finding their own solutions for the problems they face [176]. This strategy has been found to be helpful in patients with long-term physical health conditions as well [177, 178]. However, when patients are hospitalized, and avoidance behaviour is being increased, motivation for exposure therapy seems to decrease. Offering a caring environment only induces the risk of reinforcing avoidance behaviour. In these cases, it may be important to apply extrinsic motivation to foster active personal commitment [61]. This kind of restrictive care can best be offered by parents because patients need to accept the authority of their parents. However, if parents only have the opportunity to visit their child at visiting hours, an appointment with the psychiatrist or a systemic therapist, this implies parents are not important in the treatment of their child. Our view is that parents are of importance, and that the level of success in youths with chronic suicidality is dependent on the level of parental involvement [179]. This view is supported by the fact that common elements across efficacious treatments for auto-aggressive behaviour include family skills training, parent education and training, and individual skills training [180]. Furthermore, it is important not only to focus on the individual and/or parents, but also on school, friends and club life [118, 181]. Therefore, systemic interventions should not be limited to family therapy once a week, but intensively, preferably at home.

## Discussion

This essay aims to provide an integrative conceptual framework for TRIB as a guiding framework for future research and interventions. Along the HiTOP framework we outlined an aetiological model for TRIB from a transdiagnostic dimensional approach, addressing the interdependence of biologic and psychological developmental, and specific environmental aspects in relation to the development of psychopathology. The genetic and neurobiological make-up of a child and the interaction with (i.e., reaction of) parents may moderate the expression and reinforcement of emotion regulation, moral development and thus also attachment of a child. Disturbances in these interdependent developmental processes, specific attachment patterns, emotion regulation patterns and moral developmental level may be seen as higher order processes that become dominant in a way that leads to psychopathology, and as such, to an increased load on the *p* factor. Furthermore, we argue that these early developmental disturbances may lead to early social anxiety, and from thereon, to more severe psychopathology. In line with HiTOP, higher order therapeutic interventions should focus on these higher order factors that moderate psychopathology in fundamental ways. Therefore, we argue that initial therapeutic interventions in TRIB should primarily focus on restoring a secure base script and emotion regulation through intensive systemic interventions. This may help to increase adherence to therapeutic interventions in order to increase exposure and resume societal activities.

The HiTOP model identifies higher-order dimensions that reflect associations among lower-order dimensions. [13]. Although we primarily take a clinical perspective, the TRIB model seems to fit in empirical evidence regarding hierarchical dimensions for general psychopathology [182]. As such, the highest order dimension may be a single factor, the *p* factor, which is seen as the overall liability to mental disorder [10, 11], consisting of three sub-dimensions, that is, a psychotic, an internalizing and an externalizing experience dimension [52]. However, the TRIB model implies the existence of two sub-dimension, that is, a psychotic and an emotion dysregulation dimension. This is in line with previous research showing high correlations among the fears, distress, and externalizing factors [183]. Moreover, we hypothesize that an important common factor for both internalizing and externalizing behaviour may be found in common parenting practices and attachment processes. Thus, the three-factor model of psychopathology, as described by Krueger, is further condensed in the TRIB model, staying in line with the contention that mental disorders correlate because they are moderated by the same set of genetic and environmental factors [183]. Nevertheless, DSM classification may help to further tailor therapeutic approach, especially when patients are motivated.

We like to stress that the TRIB model describes *interactional* sequences between biological, psychological and social factors, and *not* a linear causal relationship in which parents are to be blamed. Nevertheless, we focus on parenting because this seems to open opportunities for positive change and increased well-being for both youths and their parents. Even though genetic and neurobiological influences may be strong [184], major interventions in the treatment of internalizing as well as externalizing behaviour primarily focus on more efficacious parenting in order to decrease externalizing behaviour. Hence, if internalizing and externalizing behaviour show similar underpinnings, and if the provision of a limit setting intervention is more effective in externalizing behaviour, what then is the best way to provide this intervention? Part of the adolescents with TRIB do not accept limit setting behaviour from their parents. Therefore, a major challenge remains in how to provide an optimal equilibrium between autonomous behaviour and restrictions on this autonomy.

The relationship with trauma, especially recurrent small-t trauma, needs further investigation, in relation to both diagnosing TRIB and treatment. Childhood rejection appears to be linked to rejection sensitivity, and rejection sensitivity has been linked to BPD [185], while high agreeableness and conscientiousness have been found to predict suicidality in relation to interpersonal trauma [186]. These findings are in line that being sensitive to rejection is related to increased levels of aggression and victimization [187], and that attachment organization has been found to be related to suicidal behaviour [188]. However, assessing developmental trauma is difficult, yet important because of increased comorbidity [189]. Hence, though small-t trauma may play an important role in the development of TRIB, further research is needed to disentangle its relationship and means for interventions.

Therapeutic alliance needs further investigation. If indeed lack of motivation for treatment is the result of lack of trust, how to increase trust? Restoration of trust through mentalization comprises an important factor in therapeutic alliance [190]. For this, solution-focused therapy (SFT) seems to provide a helpful paradigm, that is, assessing whether the patient and parents are intrinsically committed to involve and invest in treatment [191]. If this is the case, the therapeutic alliance can be labelled as a *client* relationship. If not, there are two remaining possibilities: in a *visitor* relationship the patient does not even wants to be here, in a *complainant* relationship the patient does not feel part of the problem. Both types of relationship need to be addressed accordingly to increase the chances for a positive treatment outcome. Co-construction of meaning, together with strength-oriented techniques are important in SFT [192]. Research implies SFT to be efficacious [193], with increased confidence, increased self-efficacy and increased community participation [194]. However, further research is needed on this specific labelling method regarding alliance.

The TRIB model has its limitations. As it represents a theoretical diagnostic model, it is important to investigate its diagnostic validity, such as the Robins and Guze criteria [195]. Hence, further research is needed to identify more specifically the important signs and symptoms of youth with TRIB, natural course and response to treatment. More specifically, which youths with severe emotion dysregulation are endangered to develop therapy refractoriness? As evidence is growing that environmental and contextual characteristics are inextricably linked to the underlying biological characteristics of psychopathology [196], it is important to investigate whether in this group of patients parental engagement indeed is of crucial importance. From a HiTOP perspective, it may be important to investigate whether developmental processes as emotion regulation, moral reasoning and attachment are indeed higher order dimensions moderating the *p* factor. Furthermore, is seems important to investigate whether social anxiety should be seen as higher order pathology, because it seems to represent a higher order thought disorder, that is, inflexibility in thinking, that may lead to more severe disorders.

## Summary

Auto-aggressive behaviour, especially treatment refractory suicidality in adolescents with psychiatric disorders is challenging to clinicians, especially in case there is a need for clinical treatment. This may be due to several factors in which current classification systems, based on the presence or absence of diagnostic entities, and lack of therapeutic efficacy may be important ones. In attempting to overcome these limitations, we have integrated current knowledge regarding causality and interdependency of suicidality and auto-aggressive behaviour across disorders within the HiTOP framework, in order to propose a coherent hypothetical transdiagnostic developmental framework regarding these unsettling behaviours in youths.

We argued that the interdependent development of biologic factors, attachment, moral reasoning and emotion regulation in an overprotective environment may lead to social anxiety disorder and thus to emotion dysregulation and severe internalizing behaviour disorders. Loss of trust and trauma may further moderate the development of psychopathology as well as treatment refractoriness. Avoidance behaviour and lack of motivation for treatment may appear to be the most prominent, which also may show itself in severe internalizing as well as externalizing behaviour problems. To optimize treatment efficacy for both internalizing and externalizing behaviour, it seems important to create a shift in mind-set by de-emphasizing DSM-diagnoses, addressing non-compliance, emphasizing the importance of restoration of trust between parents and their child, and limitation of avoidance behaviour.

We discussed that our model describes interactional—not linear—sequences between biological, psychological and social factors. Furthermore, it seems to be in line with the HiTOP framework, by describing higher order dimensions regarding development of psychopathology and therapy. Although this model is hypothetical, it may be helpful to increase therapeutic efficacy of both biological and psychotherapeutic interventions. Also, this model may help to provide directions for further research.
